# Eating Disorders: Assessing Its Prevalence and Pattern Among Adults With Type 2 Diabetes

**DOI:** 10.7759/cureus.52425

**Published:** 2024-01-17

**Authors:** Arti Muley, Aditi Deshmane, Anu Mahajan, Jeel Shah

**Affiliations:** 1 Nutrition and Dietetics, Symbiosis Institute of Health Sciences, Symbiosis International (Deemed University), Pune, IND

**Keywords:** ahmedabad, adults, weight concern, eating concerns, eating disorders, type 2 diabetes

## Abstract

Background: Eating disorders (EDs) are severe and multifaceted mental health issues that affect a person's perception of their body weight in relationship with food. Existing evidence shows that EDs significantly affect the physical and emotional health of individuals with Type-2 Diabetes (T2D) and are associated with impaired metabolic control and a high risk of medical complications, including higher mortality rates. However, there is a paucity of research looking into the prevalence of EDs.

Objective: A cross-sectional study was conducted to map the prevalence of EDs and to assess its pattern among adults with T2D from Ahmedabad City, Gujarat, India.

Methodology: Two hundred fifty-four T2D individuals aged 30-60 were enrolled in the study. A questionnaire was developed using the Sick, Control, One, Fat, Food (SCOFF) questionnaire and a five-question screening tool intended to identify the possibility of EDs as well as the Eating Disorder Examination-Questionnaire (EDEQ), which is used to identify the pattern of EDs with subscales like Restraint, Eating Concern, Shape Concern, and Weight Concern. Written informed consent was obtained from all participants. Descriptive statistics, Pearson’s Correlation, and Logistic Regression analysis were used. A p-value of < 0.05 was considered significant.

Results: The results revealed that 90 (35%) of the total participants were at a high risk of EDs. Among these, 54 (21% of the total population) were males, and 36 (14% of total participants) were females. There was a mild statistically significant negative correlation between age and exercise with the presence of ED (r = -0.151, p = 0.016 and r = -0.186, p = 0.003, respectively), while education showed a significant positive correlation (r = 0.150, p = 0.017). Males had significantly higher scores for eating concerns than females (19.75±4.88 vs 17.88±5.92; p = 0.008). The logistic regression model revealed that education was a significant predictor of EDs (OD = 1.47, 95% CI 1.00-2.16 and p = 0.04).

Conclusion: The study identified that people with T2D are at risk of EDs, and eating concerns worry them the most. Thus, counseling sessions should focus on identifying the determinants of EDs and educating the patients regarding quality eating. This will have implications in addressing the other morbidities as well as health risks related to BMI; especially obesity as it is more prevalent in the T2D population.

## Introduction

Eating disorders (EDs) are severe multifaceted conditions caused by persistent eating patterns that harm health, emotions, and the capacity to carry out essential life functions. Between 2000 and 2018, the prevalence of EDs rose from 3.4% to 7.8% worldwide, with the weighted means (ranges) of lifetime EDs for women being 8.4% (3.3%-18.6%) and 2.2% (0.8%-6.5%) for men. According to continents, the weighted means (ranges) of point prevalence were 4.6% (2.0%-13.5%) in America, 2.2% (0.2%-13.1%) in Europe, and 3.5% (0.6%-7.8%) in Asia [1]. It implies serious issues that impact an individual's eating habits and food-related thoughts. It is a type of mental disorder addressed in psychology, social work, nutrition, and medicine [2]. Research on the epidemiology of EDs in India has been conducted occasionally; however, these studies have all been constrained by fixed age groups and small data sets, and there have not been any countrywide studies that would allow us to determine the incidence and prevalence of EDs in India [3].

There are six types of EDs identified so far: binge ED, bulimia nervosa, anorexia nervosa, pica, avoidant restrictive food intake disorder, and rumination disorder. Pica and rumination disorder has recently been added as a new diagnostic category for EDs in the Diagnostic and Statistical Manual of Mental Disorders (DMS-5) [4]. As per the DMS-5; Pica, is craving and purposefully consuming non-food items (uncooked rice, earth), whereas rumination disorder is characterized by regurgitating recently consumed food from the stomach back to the oral cavity that too within the first 15 minutes of finishing the meal [4]. Out of these six, binge EDs with 17.3 million (11.3-24.9) and other specified feeding or EDs (OSFED) with 24.6 million (14.7-39.7) accounted for the majority of ED cases [5]. Anorexia nervosa has become the most chronic disease in adolescent girls aged 15-19 years, with a great tendency to attempt suicide [6]. A person with anorexia nervosa may communicate this acute fear of gaining weight or getting obese either directly or indirectly by engaging in behaviors that are intended to prevent it. A misunderstanding of being overweight despite being thin, an excessive influence of weight or shape on one's self-evaluation, or a failure to recognize the serious medical effects of low weight are all examples of body image disorders [7]. Similarly, Bulimia nervosa is accompanied by incorrect compensatory behavior and a significant impact of weight or shape on self-evaluation. Self-induced vomiting, laxative usage, diuretic abuse, fasting, and extreme exercise are examples of inappropriate compensatory behaviors [8].

EDs may arise from complex psychological, biological, and environmental interactions. However, chronic disease condition like diabetes is also found to play a role in developing EDs. The constant management of blood glucose levels, dietary restrictions, and concerns about complications can contribute to stress and emotional distress. Other factors such as body image concerns fear of weight gain, and the emphasis on food-related control may contribute to the development of EDs in people with diabetes [9]. Individuals with type 1 diabetes (T1D) may face a higher risk of developing EDs, particularly a condition known as “diabulimia.” According to a study, almost 93.8% of the participants with T1D and EDs stated that they were diagnosed with T1D before the diagnosis of EDs [10]. Studies suggested that the prevalence of Binge ED in type-2 diabetes (T2D) is 2% to 3.5% higher than in the general population [11,12]. EDs like Binge eating can also make it difficult for people with diabetes to control their blood glucose levels [13].

As per the special issue (Editorial) on EDs in Asia published in the International Journal of Eating Disorder [14], new prevalence and time trend data from China, Iran, Singapore, Japan, and Taiwan indicate that EDs are becoming more widespread in Asia; however, the biological risk factors are understudied and not always identified in healthcare settings. Thus, considering these facts and looking into the paucity of prevalence studies in the Indian population, the present study is designed to identify people with T2D with increased risk of EDs as well as look into the pattern and socio-demographic risk factors of EDs.

## Materials and methods

Study design

The cross-sectional study was conducted in Ahmedabad City, Gujarat, after getting approval from the Institutional Ethics Committee of Symbiosis International University Pune (No. SIU/IEC/537). A recent study done by ICMR states that the prevalence of prediabetes and diabetes in Gujarat ranges from 7.5% to 9.9% [15]. Ahmedabad city has excellent opportunities for work and business as it is one of nine metro cities in India, hence selected for our study [16]. Considering these parameters, the city is selected for the present study. The participants who met the inclusion criteria were aged 30 to 60 with known cases of T2D mellitus (T2DM) residing in Ahmedabad city. The age group was considered the sampling was purposive and getting respondents would be easy with this wide range of age. Participants excluded from the study were T1DM cases, those having any known mental disorder, pregnant or lactating women, and those who were unable to complete the questionnaire due to cognitive impairment.

Sample size calculation

It was calculated using the Population Proportion Calculator. Here, the margin of error was set at 5%, while the confidence level was set at 95%. As per the National Family Health Survey 2019-20 (NFHS-5), the fifth, the total population of Ahmedabad city is 8,651,000 people, and 40.4% of men and women have their blood sugar above normal (>140mg/dL) [17]. The proposed sample size using these values was 370 individuals, but only 254 valid responses were collected due to time and logistic constraints.

Data collection

The participants were given a brief about the objectives and purpose of the study, and written informed consent was obtained from each participant before taking their responses as an assurance that all the information they provided would be highly confidential and used only for research purposes. A modified pretested structured questionnaire was used, which included questions on the demographics, screening, and identification of ED. The study questionnaire had 32 questions and was separated into three sections: demographics, diabetes-related medical status, and screening for EDs.

Validated tools, the Sick, Control, One, Fat, Food (SCOFF) Questionnaire and Eating Disorder Examination Questionnaire (EDE-Q) were used to identify the eating pattern (Table 1). SCOFF Questionnaire is a five-question screening tool designed to determine that an ED might exist rather than to make a diagnosis. The questions can be delivered verbally or in written form. Each question is given one score, and a score of 2 or greater detects anorexia or Bulimia nervosa. Five questions of SCOFF represent a highly efficient tool for detecting EDs even by a non-specialist and is highly recommended, as shown in meta-analyses of various studies [18]. EDE-Q is a 28-item self-reported questionnaire designed to assess the range, frequency, and severity of behaviors associated with the diagnosis of an ED. EDE-Q evaluates a range of behaviors and cognitive traits associated with the disease. The latter is summed up by four subscales: shape, eating, weight, and restraint. Based on the number of days in the last 28 days or from “Not at all” to “Markedly,” items are graded on a scale of 0-6. Among these are inquiries like, “Have you ever had a real fear that you might gain weight?” and “To what extent are you unhappy with your shape?” [19]. The internal reliability of the tool was assessed using Cronbach's alpha and was acceptable at 0.75.

**Table 1:** Screening questionnaire used in the study Each yes equals 1 point; a score of 2 indicates a likely diagnosis of anorexia nervosa or bulimia. SCOFF Questionnaire [20], Eating Disorder Examination Questionaries (EDE-Q) [21].

Questionnaire	Screening questions
SCOFF questionnaire	Anacronym for the five questions used To screen primarily for anorexia and bulimia nervosa.
	Do you make yourself Sick because you feel uncomfortably full?
	Do you worry that you have lost Control over how much you eat?
	Have you recently lost more than One stone (14 lb) in 3 months?
	Do you believe yourself to be Fat when others say you are too thin?
	Would you say that Food dominates your life?
Eating Disorder Examination Questionnaire (EDE-Q)	A total of 20 questions were used to assess restraint, eating concern, shape concern, and weight concern.
	Five questions are for restrains, seven are for eating problems, three are for shape concerns, and five are for weight concerns.

Data analysis

Statistical analysis was done using Microsoft Excel and SPSS Software (Version: 20.0; IBM Corp., Armonk, NY). Descriptive analysis was used to study the demographic and medical status of the participants. Independent t-tests were used to compare the means of four subscales of EDs through which the participants' eating patterns were observed. Association between socioeconomic factors and medical status with gender was observed using a chi-square test. A p<0.05 was considered as significant. The relationship between the prevalence of EDs and socioeconomic and medical status was analyzed by Pearson’s Correlation test. Finally, Logistic Regression analysis was done to understand the predictors of EDs.

## Results

The cross-sectional study included 254 participants, among whom 55.5% (n=141) were males and 44.5% (n=113) were females. Most participants belonged to the age group of 40-50 years (54.7%) followed by 30-40 years (26.4%). The maximum number of participants were graduates (46.1%) followed by those who completed their secondary education (35.4%). It was also found that 39% of the participants exercised rarely, while 31.0% performed exercise sometimes. About 63% had normal BMI, while 15% of the male population were overweight, compared to 6% of the female population. Most participants (95%) had T2DM for less than 10 years. In addition, over 61% of the patients had uncontrolled diabetes and 70% of the population had one or more comorbidities present along with T2DM. Diabetes mellitus ran in over 60% of the participant’s family history. A significantly higher number of participants fell into the service category (40.6%) as compared to business owners (31.5%) and housewives in the case of women (28%) with p = 0.00 (Table 2).

**Table 2:** Sociodemographic information of the participants Data is presented as n, (%) BMI - Body Mass Index

Sociodemographic variables	Subgroups	Total (n=254)	Male (n=141)	Female (n=113)
Age	30 – 40 years	67 (26.4)	44 (17.4)	23 (9.1)
	40 – 50 years	139 (54.7)	69 (27.2)	70 (27.5)
	50 – 60 years	48 (18.9)	28 (11.0)	20 (7.9)
Education	Primary	43 (16.9)	20 (7.9)	23 (9.1)
	Secondary	90 (35.4)	49 (19.3)	41 (16.1)
	Graduation	117 (46.1)	71 (27.9)	46 (18.1)
	Postgraduate	4 (1.6)	1 (0.4)	3 (1.2)
Occupation	Business	80 (31.5)	73 (28.7)	7 (2.8)
	Service	103 (40.6)	68 (26.8)	35 (13.8)
	Housewife	71 (28.0)	0 (0)	71 (28.0)
Exercise	Daily	22 (8.7)	13 (5.1)	9 (3.5)
	Once/ twice a week	52 (20.5)	25 (9.8)	27 (10.6)
	Sometimes	81 (31.9)	54 (21.3)	27 (10.6)
	Rarely	99 (39.0)	49 (19.3)	50 (19.7)
BMI	Underweight	29 (11.4)	14 (5.5)	15 (5.9)
	Normal	160 (63.0)	83 (32.7)	77 (30.3)
	Overweight	53 (20.9)	37 (14.6)	16 (6.3)
	Obese	12 (4.7)	7 (2.8)	5 (1.9)
Duration of Diabetes	0 – 5 years	162 (63.8)	87 (34.3)	75 (29.5)
	6 – 10 years	83 (32.7)	48 (18.9)	35 (13.8)
	>10 years	9 (3.5)	6 (2.4)	3 (1.2)
Latest glycemic reports	Normal	99 (39.0)	52 (20.5)	47 (18.5)
	High	155 (61.0)	89 (35.0)	66 (25.0)
Medical history	No History	75 (29.5)	39 (15.4)	36 (14.2)
	Hypertension	45 (17.7)	27 (10.6)	18 (7.1)
	Thyroid	22 (8.7)	14 (5.5)	8 (3.1)
	COVID-19	51 (20.1)	29 (11.4)	22 (8.7)
	Surgeries	28 (11.0)	17 (6.7)	11 (4.3)
	More than 2	33 (13.0)	15 (5.9)	18 (7.1)
Family History of Diabetes	No	101 (39.8)	53 (20.9)	48 (18.9)
	Yes	153 (60.2)	88 (34.6)	65 (25.6)

Prevalence of ED

**Figure 1:**
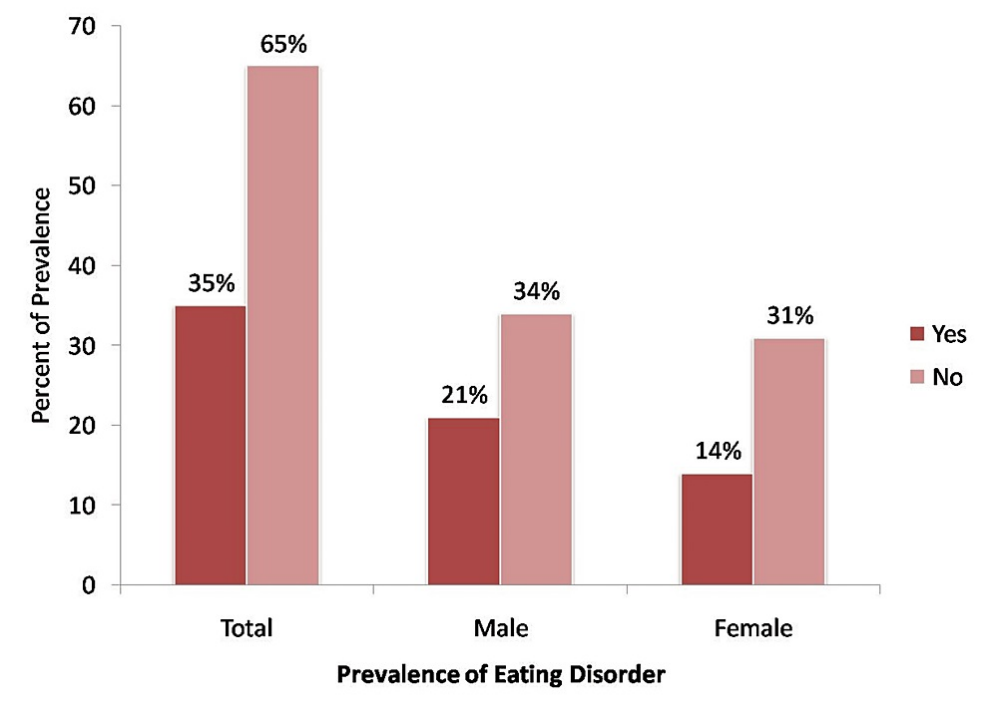
Prevalence of risk of eating disorder among the participants

As one of the study objectives was to map the prevalence of EDs, participants were asked questions to determine their eating habits. They were scored using the SCOFF questionnaire. Individuals who answered yes to more than two questions (score of 40% or more) were classified as having a high risk of developing an ED as per the criteria. Results revealed that 35% of the total individuals were at risk of developing an ED, with 21.2% being males and 14.1% being females (Figure 1).

Domain-wise assessment of ED

**Figure 2:**
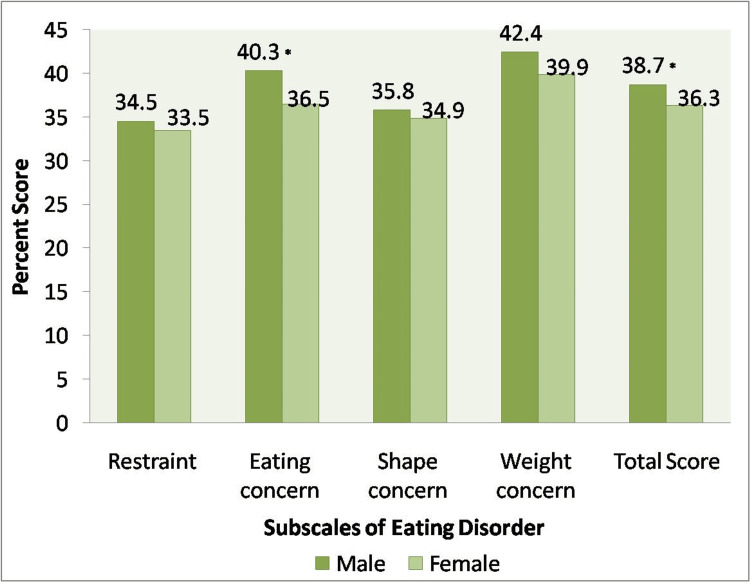
Domain-wise assessment of eating disorders *Value is significant at p<0.05

An independent sample t-test results suggest potential gender-specific patterns in the aspects of EDs. Based on the responses to EDE-Q, scores were obtained for the four subscales: Restraints, Eating Concern, Shape Concern, and Weight Concerns. A statistically significant difference existed in total scores between males (38.70 ± 7.96) and females (36.32 ± 10.55) with p = 0.042. Similarly, a statistically significant difference existed in eating concern scores between males (40.30 ± 9.96) and females (36.49 ±12.08) at p = 0.006 as well (Figure 2). These findings highlight the importance of considering gender-specific factors when examining eating behavior patterns.

Correlation of ED with sociodemographic variables

The correlation analysis investigated the association between the prevalence of EDs and various demographic and health-related factors. It was seen that there is a mild statistically significant negative correlation between age and the presence of EDs (r = -0.151, p = 0.016). This suggests that, on average, as age decreases, there is an increase in the likelihood of EDs. Similarly, the exercise frequency also showed a statistically significant negative correlation with ED presence (r = -0.186, p = 0.003), suggesting that participants who are more likely to exercise are less likely to report the presence of EDs. Lastly, a mild statistically significant positive correlation existed between education and ED presence (r = 0.150, p = 0.017). This suggests that individuals with higher education levels (Graduates) may be more likely to report the presence of ED (Table 3).

**Table 3:** Correlation of eating disorder with socioeconomic and medical variables Data is presented as N, (%) *Value is significant at p<0.05 BMI - Body Mass Index

Sociodemographic Variables	Subgroups	High risk of eating disorder (N=254)		p-value
		No (N = 164)	Yes (N = 90)	
Gender	Male	87 (53.1%)	54 (60%)	0.288
	Female	77 (46.9%)	36 (40%)	
Age	30 – 40 years	40 (24.4%)	27 (30%)	0.016*
	40 – 50 years	84 (51.2%)	55 (61.1%)	
	50 – 60 years	40 (24.4%)	8 (8.9%)	
Education	Primary	32 (19.5%)	11 (12.3%)	0.01*
	Secondary	65 (39.6%)	25 (27.7%)	
	Graduation	63 (38.4%)	54 (60%)	
	Post graduate	4 (2.4%)	0 (0%)	
Occupation	Business	52 (31.7%)	28 (31.1%)	0.759
	Service	64 (39.0%)	39 (43.3%)	
	Housewife	48 (29.3%)	23 (25.5%)	
Exercise	Daily	17 (10.4%)	5 (5.5%)	0.003*
	Once/ twice a week	43 (26.2%)	9 (10%)	
	Sometimes	47 (28.6%)	34 (37.8%)	
	Rarely	57 (34.7%)	42 (46.6%)	
BMI	Underweight	18 (11.0%)	11 (12.2%)	0.706
	Normal	106 (64.4%)	54 (60%)	
	Overweight	33 (20.3%)	20 (22.2%)	
	Obese	7 (4.3%)	5 (5.5%)	
Duration of Diabetes	0 – 5 years	103 (62.8%)	59 (65.5%)	0.374
	6 – 10 years	53 (32.3%)	30 (33.3%)	
	>10 years	8 (4.9%)	1 (1.1%)	
Latest blood reports	Normal	69 (42.0%)	30 (33.3%)	0.173
	High	95 (58.0%)	60 (66.6%)	
Co-morbidity	No co-morbidity	53 (32.2%)	22 (24.4%)	0.374
	Hypertension	30 (18.3%)	15 (16.6%)	
	Thyroid	10 (6.1%)	12 (13.3%)	
	COVID-19	32 (19.5%)	19 (21.1%)	
	Surgeries	18 (10.1%)	10 (11.1%)	
	>2 comorbidities	21 (12.8%)	12 (13.3%)	
Family History of Diabetes	No	66 (40.2%)	35 (38.9%)	0.834
	Yes	98 (59.8%)	55 (61.1%)	

Logistic regression to find predictors for the prevalence of EDs

The logistic regression analysis was performed to identify which variables among gender, age, BMI, education, occupation, medical history of co-morbidities, family history of diabetes, duration of diabetes, and glycemic control would predict the prevalence of EDs. Education was a significant predictor of EDs (OD = 1.47, 95% CI 1.00-2.16 and p = 0.04). For each one-unit increase in education, the odds of having an ED increased by approximately 47.2% (Table 4). These findings contribute to our understanding of factors associated with EDs and may inform targeted intervention strategies.

**Table 4:** Logistic regression to predict prevalence of eating disorder *Value is significant at p<0.05 BMI - Body Mass Index

Predictor	Coefficient	Standard Error	P-value	Odds Ratio	95% CI Lower	95% CI Upper
Gender	-0.468	0.387	0.227	0.626	0.255	1.526
Age	-0.400	0.222	0.072	0.671	0.446	1.008
BMI	0.120	0.199	0.545	1.128	0.756	1.682
Education	0.386	0.195	0.048*	1.472	1.001	2.160
Occupation	0.254	0.259	0.326	1.289	0.803	2.073
Medical history	0.119	0.079	0.130	1.127	0.965	1.318
Duration of Diabetes	-0.074	0.260	0.777	0.929	0.525	1.646
Family History of Diabetes	0.025	0.281	0.930	1.025	0.529	1.985
Glycemic Control	0.280	0.285	0.327	1.323	0.775	2.266

## Discussion

The present study aimed to assess the risk of EDs among T2DM, and it was observed that 35% of the total population had a high risk of EDs. This finding is high compared to cross-sectional study screening for positive EDs. According to this study, 14% of the participants screened positive for EDs [22]. Other studies found a varying prevalence of 40% and 20% [23,24]. This difference in the prevalence of ED among T2DM individuals can be due to different screening tools, sample characteristics, and inclusion criteria. Among all the participants, 21% of males and 14% of females were at high risk of EDs, unlike one study which found the prevalence in 19.6% of women while 10.2% of men [25]. A similar trend of females having a higher risk of EDs than males was seen in yet another study [26]. However, one study found that males had a significantly higher frequency of ED than females, similar to our study findings [27].

There was a mild negative significant association between EDs among different age groups, which states that elders are less prone to EDs. This negative correlation with age found in our study breaks away with existing literature, indicating that it tends to be more prevalent in older individuals or women during their menopausal period reported to have extreme EDs [28]. Demographic variables like education positively correlated and were a statistically significant predictor of the prevalence of EDs. Individuals who were graduates showed the highest prevalence of EDs as compared to participants who had primary or secondary education. This positive correlation with education contradicts a study finding that investigated the unclassifiable EDs and found the association the other way around [29], and further investigation may be warranted to explore the underlying factors contributing to this association. Similarly, a correlation between exercise and EDs was also seen in our study. The negative correlation between exercise frequency and EDs presence raises intriguing questions about the interplay between physical activity and EDs. Demographics like gender, BMI, occupation, duration of diabetes, and the presence of medical and family history did not correlate.

Lastly, looking into the pattern of EDs, it was seen that eating concern was the second most in all the four subscales and was statistically high in males compared to females. The most crucial component of glycemia control is proper food management. Dietary advice should be flexible rather than rigorous because rigidity can lead to episodes of loss of control and binge eating. A restricted diet combined with restrictive beliefs about weight, food, and drugs may increase the likelihood and frequency of binges [30]. Weight loss as a therapy goal for overweight or obese patients with T2DM should be reconsidered since disordered eating behaviors must be resolved before weight loss is pursued. The probability and fear of weight gain must be discussed because improved adherence to diabetes treatment may result in subsequent weight gain, so patients must be aware that weight gain that follows improved metabolic control is caused by water retention rather than an increase in body fat. It is necessary to assess and analyze dietary satisfaction and barriers.

Strengths and limitations of the study

Assessing the prevalence of EDs among the T2D population is essential as the co-occurrence of EDs and T2D can have profound health implications like poor glycemic control, increased risk of hypoglycemia and ultimately the poor quality of life. Recognizing and addressing EDs is also crucial for providing patient-centered care. The present study highlights the prevalence and can help healthcare professionals tailor treatment plans as patients with co-morbid EDs and T2D may require specialized interventions that address both conditions simultaneously. This study also helps identify the risk factors of developing EDs and the pattern; this will help proactive intervention. Insights gained from such studies can also inform future research and clinical practices.

However, our study has a few limitations as well. Firstly, as the study was cross-sectional, there are limitations to generalizing findings to different periods or populations and causal inference. Secondly, as per the calculated sample size, about 31% could not be met due to constraints. Lastly, there is a possibility of self-reporting bias on the part of participants.

## Conclusions

In T2DM individuals, EDs assessment should include questions regarding body image and structure, as well as binge eating and purging behavior. Clinicians should pay close attention to possible diabetes medication manipulation in T2DM people, as well as the motivations for modifications or non-adherence to treatment. The use of standardized evaluation tools in conjunction with clinical interviews can help these patients be screened for disordered eating behavior.

While there is a growing body of research on the link between EDs and diabetes, there is still much to learn. Future research should focus on developing effective prevention and treatment strategies for individuals with diabetes and EDs. The use of technology, such as mobile apps, artificial intelligence (AI), and telehealth may also help manage EDs and diabetes. These tools can provide individuals with real-time feedback and support, making it easier to manage their conditions. It is also vital to continue raising awareness of the link between EDs and diabetes among healthcare professionals and the public. Increasing awareness can help prevent and treat these conditions effectively and improve the quality of life for individuals affected by them. The study will also contribute to the existing literature on EDs among individuals with diabetes, as there are very few studies conducted on individuals with T2D.
